# Comparative Pharmacokinetics of Levofloxacin in Healthy and Renal Damaged Muscovy Ducks following Intravenous and Oral Administration

**DOI:** 10.1155/2014/986806

**Published:** 2014-02-04

**Authors:** Mohamed Aboubakr, Ahmed Soliman

**Affiliations:** ^1^Department of Pharmacology, Faculty of Veterinary Medicine, Benha University, Moshtohor, Toukh, Qaliobiya 13736, Egypt; ^2^Department of Pharmacology, Faculty of Veterinary Medicine, Cairo University, Giza 12211, Egypt

## Abstract

The pharmacokinetics aspects of levofloxacin were studied in healthy and experimentally renal damaged Muscovy ducks after single intravenous (IV) and oral (PO) dose of 10 mg kg^−1^ bwt. Following IV administration, elimination half-life (*t*_1/2(**β**)_) and mean residence time (MRT) were longer in renal damaged ducks than in healthy ones. Total clearance (Cl_tot_) in renal damaged ducks (0.20 L kg^−1^ h^−1^) was significantly lower as compared to that in healthy ones (0.41 L kg^−1^ h^−1^). Following PO administration, the peak serum concentration (*C*_max_) was higher in renal damaged than in healthy ducks and was achieved at maximum time (*t*_max_) of 2.47 and 2.05 h, respectively. The drug was eliminated (*t*_1/2(el)_) at a significant slower rate (3.94 h) in renal damaged than in healthy ducks (2.89 h). The pharmacokinetic profile of levofloxacin is altered in renal damaged ducks due to the increased serum levofloxacin concentrations compared with that in clinically healthy ducks. Oral administration of levofloxacin at 10 mg kg^−1^ bwt may be highly efficacious against susceptible bacteria in ducks. Also, the dose of levofloxacin should be reduced in renal damaged ducks. Pharmacokinetic/pharmacodynamic integration revealed significantly higher values for *C*_max_/MIC and AUC/MIC ratios in renal damaged ducks than in healthy ones, indicating the excellent pharmacokinetic characteristics of levofloxacin in renal damaged ducks.

## 1. Introduction

Poultry industry has developed tremendously in the last two decades. Although a major breakthrough in poultry production had been made during the last years, the problems facing this industry are many and diverse. It is also seen that in a number of avian diseases, vital organs like the liver and kidney become affected. The kidney is the main route of drug elimination and removal of the drugs from the body by excretion. Under these conditions, the drugs of choice, route of administration, and dosage regimen of the efficient drugs are not clearly known and literature is unavailable that might have dictated the indiscriminate and unscientific use of antibiotics leading to economic loss, and as a consequence poultry birds and poultry farmers suffer a lot [1].

Levofloxacin is a third-generation fluoroquinolone that possesses activity against most aerobic Gram-positive and Gram-negative organisms and demonstrates moderate activity against anaerobes [2], as well as atypical pathogens such as *Mycoplasma *and* Chlamydia* [3]. The bactericidal effect of levofloxacin is achieved through reversible binding to DNA gyrase and subsequent inhibition of bacterial DNA replication and transcription [4]. Levofloxacin distributes well to target body tissues and fluids in the respiratory tract, skin, urine, and prostrate, and its uptake by cells makes it suitable for use against intracellular pathogens. The drug undergoes limited metabolism in rats and human [5] and is primarily excreted by kidney mainly as active drug. Inactive metabolites (N-oxide and demethyl metabolites) represent <5% of the total dose [6].

The pharmacokinetics of levofloxacin has been investigated in many animal species including rabbits [7], cats [8], calves [9], stallions [10], camels [11], lactating goats [12], and quails [13]. However, there is no available information on the kinetics of levofloxacin in Muscovy ducks and the literature in respect to pharmacokinetics in healthy and diseased states is scarcely available. The present research work was carried out to study the disposition kinetics of levofloxacin in healthy ducks and its modification in renal damaged ones following a single IV and PO administration of 10 mg kg^−1^ bwt. Based on its pharmacological profile, levofloxacin could be a promising therapeutic tool for several infections in ducks.

## 2. Materials and Methods

### 2.1. Drugs and Chemicals

Tavanic [100 mL vial of solution of levofloxacin hemihydrate equivalent to 500 mg (5 mg/mL) levofloxacin] and levofloxacin oral tablets (Tavanic 500 mg) were purchased from Sanofi-Aventis, Pharmaceutical Ltd., Egypt, and Mueller-Hinton agar was purchased from Mast Group Ltd., Merseyside, UK.

### 2.2. Experimental Birds

Eighteen clinically healthy male Muscovy ducks, 15 weeks old, weighing between 4 and 4.5 kg, were bought from a commercial farm. Birds were housed under control conditions (at 25°C) and fed antibacterial free balanced commercial rations and drinking water was freely available. During acclimatization (at least two weeks before starting the experiment to ensure the complete withdrawal of any residual drugs) and subsequent treatment periods, their health status was checked by daily observations and no clinical signs of disease were seen. The experiment was performed in accordance with the guidelines set by the Ethical Committee of Benha University, Egypt.

### 2.3. Experimental Design

Ducks were individually weighed before drug administration and doses were calculated precisely. Ducks were divided into three groups of 6 each. The first group was considered as control (healthy), while 2nd and 3rd groups were made renal damaged following IV administration of uranyl nitrate 2 mg kg^−1^, for 4 consecutive days [1]. Blood urea nitrogen (BUN), creatinine, and uric acid levels were determined calorimetrically using kits from Diamond Diagnostic Company (Egypt), to assess the intensity of kidney damage. The dose level of uranyl nitrate 2 mg kg^−1^ for inducing renal damage was determined from the pilot studies [1].

Clinically healthy ducks were given levofloxacin 10 mg kg^−1^ as a single IV dose (through the wing vein) and a single PO dose with a 2-week washout period between each route. The renal damaged ducks within the 2nd group were given the drug intravenously into the right wing vein while the 3rd group was given the drug orally. Levofloxacin was administered after 4 days of uranyl nitrate administration in 2nd and 3rd groups. A vein flow catheter was introduced into the left wing vein of the birds and fixed with adhesive tape. Blood samples (1 mL) were collected through the vein flow catheter of the birds from the three groups immediately prior to medication (time = 0) and then at 0.08, 0.17, 0.25, 0.5, 0.75, 1, 2, 4, 6, 8, 10, 12, 18, 24, 30, and 48 h after treatment, from the left wing vein, into tubes containing heparin. Plasma was separated after centrifugation at 3,000 g for 15 minutes. The plasma was decanted, labeled, and frozen at −20°C until the assays were performed.

### 2.4. Analytical Method

The concentration of levofloxacin in plasma samples was estimated by a standard microbiological assay using *Escherichia coli* ATCC 10536 as test microorganism [14]. This method estimated the level of drug having antibacterial activity, without differentiating between the parent drug and its active metabolites. The application of microbiological assay for measuring levofloxacin concentration is suitable [8]. Standard curves were constructed using antibacterial free plasma collected from ducks. The wells were filled with 100 *μ*L of either the test samples or levofloxacin standards. The plates were kept at room temperature for 2 h before being incubated at 37°C for 18 h. Zones of inhibition were measured using micrometers, and the levofloxacin concentrations in the test samples were calculated from the standard curve. The calibration curves of plasma were prepared with different concentrations between 0.05 and 25 *μ*g/mL using blank Muscovy ducks plasma. The limit of quantification (LOQ) was 0.05 *μ*g/mL of levofloxacin in supplemented duck plasma. Under our experimental conditions, the linearity of the method was from 0.05 to 25 *μ*g/mL of levofloxacin duck plasma, and the value of correlation coefficients (*r*) was 0.991. The precision and accuracy of the method were evaluated by repetitive analysis of the plasma samples (*n* = 12) spiked with different known concentrations of levofloxacin. Intra-assay variations were determined by measuring six replicates (*n* = 6) of three standard samples used for calibration curves. The intra-assay variation coefficients were <4.87%. Interassay precisions were determined by assaying the three standard samples on three separate days. The Interassay variation coefficients were <4.46%. Recovery of levofloxacin from plasma was found to be 91.56 ± 2.11%.

### 2.5. Pharmacokinetic Analysis

Following IV administration, the plasma concentrations versus time data of the drug in healthy and renal damaged ducks were fitted to a two-compartment open model system according to the following biexponential equation [15]:

(1) $$\begin{array}{c} C_{p}=Ae^{-\alpha t}+Be^{-\beta t}, \end{array}$$

where *C*_*p*_ is the concentration of drug in the plasma at time *t*, *A* and *B* are the zero-time drug intercepts of the distribution and elimination phase expressed as *μ*g mL^−1^, *α* and *β* are the distribution and elimination rate constants expressed in units of reciprocal time (h^−1^), and e is the natural logarithm base.

A computerized program WinNonlin 4.1 (Pharsight, Mountain View, CA, USA) was used to analyze the concentration-time curves for each individual duck after the administration of levofloxacin by different routes. For the IV data, the appropriate pharmacokinetic model was determined by visual examination of individual concentration-time curves and by application of Akaike's Information Criterion (AIC) [16]. The volume of distribution at steady state (Vd_ss_), the total body clearance (Cl), and mean residence time (MRT) were computed according to standard equations [17]. Following PO administration, plasma concentration data in healthy and renal damaged ducks were analyzed by compartmental and noncompartmental methods based on the statistical moment theory [17]. In compartmental analysis, best fitting of the data was accomplished using the one-compartment open model. The area under the concentration-time curve (AUC) and area under the first moment curve (AUMC) were calculated by the method of trapezoids. Mean residence time (MRT) was calculated as MRT = AUMC/AUC and the systemic clearance as Cl = Dose/AUC. The absolute bioavailability was calculated as *F* = AUC_PO_/AUC_IV_ × 100. Mean absorption time was calculated as MAT = MRT_po_ − MRT_IV_. The pharmacokinetic parameters were reported as mean ± SE. Mean pharmacokinetic parameters after IV and PO administrations were statistically compared in healthy and renal damaged ducks using Student's *t*-test [18].

## 3. Results

**Figure 1:**
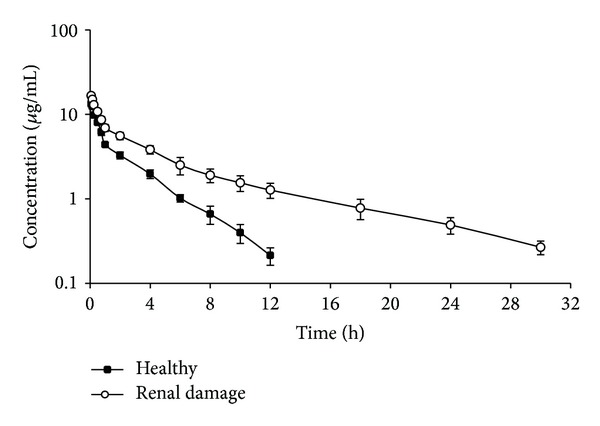
Semilogarithmic plot of the observed mean ± SE depicting the time and concentration of levofloxacin in plasma of healthy (■) and renal damaged (*о*) ducks after a single IV administration of 10 mg kg^−1^ bwt (*n* = 6).

**Figure 2:**
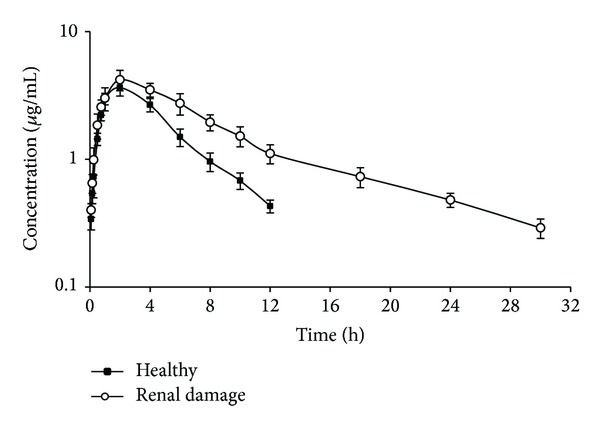
Semilogarithmic plot of the observed mean ± SE depicting the time and concentration of levofloxacin in plasma of healthy (■) and renal damaged (*о*) ducks after a single PO administration of 10 mg kg^−1^ bwt (*n* = 6).

Clinical examination of all ducks before and after each trial did not reveal any abnormalities. No local or adverse reactions to levofloxacin occurred after IV and PO administrations. The mean plasma concentration-time profiles of levofloxacin following single IV and PO administrations of 10 mg kg^−1^ bwt in both healthy and renal damaged ducks were presented graphically in Figures 1 and 2. The BUN, creatinine, and uric acid levels were gradually increased in 2nd and 3rd groups. BUN was significantly increased from 27.85 to 59.34–63.41 mg dL^−1^, creatinine level was significantly increased from 1.08 to 3.29–4.12 mg dL^−1^, and uric acid level was significantly increased from 4.56 to 8.97–9.75 mg dL^−1^ at 0 and 4th days, respectively, after daily IV administration of uranyl nitrate at 2 mg kg^−1^ for 4 consecutive days. Levofloxacin could not be detected in plasma beyond a 12 h period in healthy ducks and a 30 h in period renal damaged ducks. It was also observed that the concentrations of levofloxacin were significantly higher in all the samples of renal damaged ducks compared to healthy ones. Pharmacokinetics parameters estimated from the curve fitting following IV and PO administrations were shown in Tables 1 and 2. Renal damage changed the profile of levofloxacin pharmacokinetics as seen in the increased AUC, prolonged half-life, and MRT and decreased both volume of distribution (Vd_ss_) and total body clearance (Cl_tot_).

## 4. Discussion

The present investigation revealed that the elimination half-life (*t*_1/2(*β*)_) of levofloxacin in healthy ducks following IV administration was 2.75 h. This observation agreed with the data reported for levofloxacin in stallions 2.58 h [10], camels 2.92 h [11], and quails 2.52 h [13] and for marbofloxacin in Muscovy ducks 2.83 h [19] and longer than that reported in calves 1.61 h [9] and shorter than that reported in rabbits 7.5 h [7]. The *k*_12_/*k*_21_ ratio was 1.09, indicating a faster drug transportation rate from the central to the peripheral compartment than redistribution from the peripheral to the central compartment. This value agreed with the data reported for levofloxacin (1.13) in lactating goats [12].

The Vd_ss_ of levofloxacin in healthy ducks was 1.37 L kg^−1^, suggesting good penetration through biological membranes and tissue distribution after IV administration. This value is similar to those values reported for orbifloxacin 1.17 L kg^−1^, for marbofloxacin 1.25 L kg^−1^ in ducks, respectively, [20, 21] and for levofloxacin in quails 1.27 L kg^−1^ [13], longer than that reported in lactating goats 0.73 L kg^−1^ [12], and shorter than that reported for danofloxacin in ducks 5.41 L kg^−1^ [22]. The total body clearance (Cl_tot_) was 0.41 L kg^−1 ^h^−1^; these results agreed with the data reported in ducks, for moxifloxacin 0.32 L kg^−1 ^h^−1^ [23], higher than that for marbofloxacin 0.16 L kg^−1 ^h^−1^ [19] and lower than that for danofloxacin 1.01 L kg^−1 ^h^−1^ [22]. The high value of AUC (24.54 *μ*g mL^−1 ^h^−1^) reflects that a vast area of the body is covered by drug concentration. Similar to the present study, high values of AUC of levofloxacin have also been reported in rabbits 29.7 *μ*g mL^−1 ^h^−1^ [7], lactating goats 23.94 *μ*g mL^−1 ^h^−1^ [12], quails 24.03 *μ*g mL^−1 ^h^−1^ [13] and for orbifloxacin in ducks 26.2 *μ*g mL^−1 ^h^−1^ [20].

It was found that the value of Cl_tot_ (0.20 L kg^−1 ^h^−1^) in renal damaged ducks was significantly (*P* < 0.05) different as compared to healthy ones. The fact that clearance (Cl) is a function, whose value depends upon volume of distribution, and that this parameter decreased in renal damaged conditions could be the reason for decreased Cl value of levofloxacin in renal damaged ducks. The renal clearance of drug is blood flow dependent, so the elimination by the kidney can be impaired when reduced cardiac output compromises renal blood flow [24]. Renal damage produces some functional changes including a decrease in renal blood flow and glomerular filtration rate, which induced an increase in elimination half-life and decrease in plasma and renal clearance [25]. The clearance of marbofloxacin in dogs was slightly decreased after the induction of renal failure [26].

Following PO administration, the mean plasma concentrations of levofloxacin were significantly higher in renal damaged ducks, consistent with a long elimination half-life in diseased ducks (*t*_1/2(el)_ = 3.94 h) as compared with the value for healthy ones 2.89 h. Levofloxacin was rapidly and efficiently absorbed through gastrointestinal tract of healthy Muscovy ducks as the absorption half-life (*t*_1/2(ab)_ = 0.21 h). The obtained value was shorter than marbofloxacin in ducks 0.34 h [19]. The rapid oral absorption was also reflected by low MAT (mean absorption time) value 0.31 h. This value was shorter than danofloxacin in ducks 1.01 h [22]. The elimination half-life (*t*_1/2(el)_) was 2.89 h; this observation was lower than the data reported for orbifloxacin in ducks 4.18 h [20] and for marbofloxacin in ducks 4.61 h [21] but higher than that for moxifloxacin in chickens 1.69 h [27]. Maximal plasma concentration (*C*_max⁡_) was 3.63 *μ*g/mL achieved at (*T*_max⁡  _) 2.05 h. These values were higher than those for marbofloxacin in ducks 1.13 *μ*g/mL at 1.41 h [21] and lower than those for pefloxacin in chicken 3.78 *μ*g/mL at 3.33 h [28]. Bioavailability is the fraction of a drug administered by any nonvascular route that gains access to the systemic circulation. Following PO administration, the systemic bioavailability of levofloxacin in healthy ducks was 73.56% which is almost the same with oral bioavailability reported for marbofloxacin in ducks 72.35% [19].

The elimination half-life of levofloxacin following IV and PO administrations was longer in renal damaged ducks than healthy ones. This delay in the elimination of the drug may be the result of renal abnormalities caused by uranyl nitrate; this result agreed with [29], that observed the prolongation in the elimination half-life of ciprofloxacin in uranyl nitrate-treated rats when compared with those in the normal rats. A longer mean residence time (MRT) was found in renal damaged ducks as compared to healthy ones. The discrepancies between values calculated for pharmacokinetic parameters may be attributed to the animal species, the drug formulation employed, the age, size, or sex of the animals, to differences in fatty tissue deposits between animal species or breeds, or even to interindividual variations and also due to the method of analysis of the drug [30]. Apparent differences were observed in pharmacokinetics parameters of ciprofloxacin between patients with severe impairment of renal function and those with normal renal function, AUC was increased, Cl was reduced, and *t*_1/2(*β*)_ was prolonged in impaired renal function patients [31]. Also, in uranyl nitrate-treated rats, Cl was reduced compared with those in the normal rats [29].

Based on many *in vitro* and *in vivo* studies performed in humans and animals, it has been established that for concentration dependant antibacterial agents, such as fluoroquinolones, the AUC/MIC ratio is the most important factor in predicting efficacy, with the rate of clinical cure being greater than 80%, when this ratio is higher than 100–125 [32–34]. A second predictor of efficacy for concentration dependent antibiotic is the ratio *C*_max⁡_/MIC, considering that values above 8–10 would lead to better clinical results and to avoidance of bacterial resistance emergence [33–37]. Levofloxacin pharmacokinetic/pharmacodynamic integration revealed significantly higher values for *C*_max⁡_/MIC and AUC/MIC ratios in renal damaged ducks than in healthy ducks, indicating the excellent pharmacokinetic characteristics of the drug in renal damaged ducks. The pharmacokinetics of levofloxacin in healthy ducks, and renal damaged ones, was significantly different: the clearance had a lower mean and a higher variance in renal damaged than in healthy ducks, and the concentrations of levofloxacin were higher when renal damage was established. These differences can be expected to optimize efficacy and minimize the development of resistance. The MIC of levofloxacin has not yet been determined for bacteria isolated from ducks. To cover most of the susceptible organisms, in this discussion, the MIC_90_ of 0.032–0.5 *μ*g/mL was reported as minimum therapeutic concentration (MIC_90_) for levofloxacin against most bacteria [38]. An average MIC_90_ of 0.1 *μ*g/mL of levofloxacin has been taken into consideration for calculation of efficacy predictors. Following PO administration in healthy and renal damaged ducks, the *C*_max⁡_/MIC ratio of 36.29 and 40.52 and AUC/MIC ratio of 179.72 and 373.81, respectively, indicates potential clinical and bacteriological efficacy of levofloxacin in ducks.

## 5. Conclusion

These data allow the conclusion that levofloxacin administered intravenously and orally to ducks at a dose rate of 10 mg kg^−1^ bwt could be useful in the treatment of bacterial infections that cause renal damage in ducks.

## Figures and Tables

**Table 1:** Pharmacokinetic parameters of levofloxacin in healthy and renal damaged ducks after a single IV administration of 10 mg kg^−1^ bwt (*n* = 6).

Parameter	Unit	Healthy	Renal damaged
*C* ^*o*^	*μ*g mL^−1^	15.27 ± 1.08	18.33 ± 0.56*
*A *	*μ*g mL^−1^	10.20 ± 0.60	11.51 ± 0.40
*B *	*μ*g mL^−1^	5.05 ± 0.5	6.81 ± 0.4*
*α*	h^−1^	2.35 ± 0.19	2.09 ± 0.21
*β*	h^−1^	0.25 ± 0.01	0.15 ± 0.02**
*K*_12_	h^−1^	1.03 ± 0.11	1.01 ± 0.11
*K*_21_	h^−1^	0.94 ± 0.08	0.87 ± 0.09
*K*_12_/*K*_21_	Ratio	1.09 ± 0.04	1.17 ± 0.06
*t*_1/2(*α*)_	h	0.30 ± 0.02	0.33 ± 0.04
*t*_1/2(*β*)_	h	2.76 ± 0.10	4.71 ± 0.54**
Vd_ss_	L kg^−1^	1.37 ± 0.07	1.18 ± 0.04*
Cl_(tot)_	L kg^−1^h^−1^	0.41 ± 0.04	0.20 ± 0.02*
AUC	*μ*g mL^−1^h^−1^	24.43 ± 2.46	52.01 ± 8.34*
AUMC	*μ*g mL^−1^h^−2^	81.81 ± 12.21	323.55 ± 94.57*
MRT	h	3.34 ± 0.16	6.13 ± 0.76**

*P < 0.05, **P < 0.01.

*C* ^*o*^: concentration at zero time (immediately after single IV administration); *A*, *B*: zero-time intercepts of the biphasic disposition curve; *α*, *β*: hybrid rate constants representing the slopes of distribution and elimination phases, respectively; *K*_12_: first-order constant for transfer from central to peripheral compartment; *K*_21_: first-order constant for transfer from peripheral to central compartment; *t*_1/2(*α*)_: distribution half-life; *t*_1/2(*β*)_: elimination half-life; Vd_ss_: volume of distribution at steady state; Cl_(tot)_: total body clearance; AUC: area under serum concentration-time curve; AUMC: area under moment curve; MRT: mean residence time.

**Table 2:** Pharmacokinetic parameters of levofloxacin in healthy and renal damaged ducks after a single PO administration of 10 mg kg^−1^ bwt (*n* = 6).

Parameter	Unit	Healthy	Renal damaged
*K*_ab_	h^−1^	3.31 ± 0.10	0.47 ± 0.07***
*K*_el_	h^−1^	0.24 ± 0.02	0.18 ± 0.01*
*t*_1/2(ab)_	h	0.22 ± 0.01	1.45 ± 0.08***
*t*_1/2(el)_	h	2.89 ± 0.09	3.94 ± 0.14***
*C*_max⁡_	*μ*g mL^−1^	3.63 ± 0.12	4.05 ± 0.13
*t*_max⁡_	h	2.05 ± 0.08	2.47 ± 0.11*
AUC	*μ*g mL^−1^h^−1^	17.97 ± 2.24	37.38 ± 2.28***
AUMC	*μ*g mL^−1^h^−2^	37.46 ± 2.61	255.49 ± 17.59***
MRT	h	4.08 ± 0.14	6.83 ± 0.19***
MAT	h	0.31 ± 0.08	2.08 ± 0.07***
*F *	%	73.56 ± 2.38	71.88 ± 2.42
*C*_max⁡_/MIC	Ratio	36.29 ± 2.44	40.52 ± 2.47
AUC/MIC	Ratio	179.72 ± 11.35	373.81 ± 21.03***

*P < 0.05, ***P < 0.001.

*k*_ab_: first-order absorption rate constant; *K*_el_: elimination rate constant; *t*_1/2(ab)_: absorption half-life; *t*_1/2(el)_: elimination half-life; *C*_max⁡_: maximum plasma concentration; *t*_max⁡_: time to peak plasma concentration; MAT: mean absorption time; *F*: fraction of drug absorbed systemically after PO administration; *C*_max⁡_/MIC: maximum serum concentration/minimum inhibitory concentration ratio; AUC/MIC: area under concentration-time curve/MIC ratio.
