# Erratum to “Comparative Pharmacokinetics of Levofloxacin in Healthy and Renal Damaged Muscovy Ducks following Intravenous and Oral Administration”

**DOI:** 10.1155/2014/390875

**Published:** 2014-03-16

**Authors:** Mohamed Aboubakr, Ahmed Soliman

**Affiliations:** ^1^Department of Pharmacology, Faculty of Veterinary Medicine, Benha University, Moshtohor, Toukh, Qaliobiya 13736, Egypt; ^2^Department of Pharmacology, Faculty of Veterinary Medicine, Cairo University, Giza 12211, Egypt

The following errors occurred in Table 2 and Page 4.

AUMC value in healthy ducks is 73.46 *μ*g mL^−1^ h^−2^ instead of 37.46 *μ*g mL^−1^ h^−2^.

MAT value in healthy ducks is 0.74 h instead of 0.31 h and MAT value in renal damaged ducks is 0.70 h instead of 2.08 h, with no significance in MAT between healthy and renal damaged ducks.

In Page 4, last line in left column, low MAT (mean absorption time) value is 0.74 h instead of 0.31 h.

## Figures and Tables

**Table 2:** Pharmacokinetic parameters of levofloxacin in healthy and renal damaged ducks after a single PO administration of 10 mg kg^−1^ bwt (*n* = 6).

Parameter	Unit	Healthy	Renal damaged
*K*_ab_	h^−1^	3.31 ± 0.10	0.47 ± 0.07***
*K*_el_	h^−1^	0.24 ± 0.02	0.18 ± 0.01*
*t*_1/2(ab)_	h	0.22 ± 0.01	1.45 ± 0.08***
*t*_1/2(el)_	h	2.89 ± 0.09	3.94 ± 0.14***
*C*_max⁡_	*μ*g mL^−1^	3.63 ± 0.12	4.05 ± 0.13
*t*_max⁡_	h	2.05 ± 0.08	2.47 ± 0.11*
AUC	*μ*g mL^−1^h^−1^	17.97 ± 2.24	37.38 ± 2.28***
AUMC	*μ*g mL^−1^h^−2^	73.46 ± 2.61	255.49 ± 17.59***
MRT	h	4.08 ± 0.14	6.83 ± 0.19***
MAT	h	0.74 ± 0.08	0.70 ± 0.07
*F *	%	73.56 ± 2.38	71.88 ± 2.42
*C*_max⁡_/MIC	Ratio	36.29 ± 2.44	40.52 ± 2.47
AUC/MIC	Ratio	179.72 ± 11.35	373.81 ± 21.03***

*P < 0.05, ***P < 0.001 refers to degree of significance.

*k*_ab_: first-order absorption rate constant; *K*_el_: elimination rate constant; *t*_1/2(ab)_: absorption half-life; *t*_1/2(el)_: elimination half-life; *C*_max⁡_: maximum plasma concentration; *t*_max⁡_: time to peak plasma concentration; MAT: mean absorption time; *F*: fraction of drug absorbed systemically after PO administration; *C*_max⁡_/MIC: maximum serum concentration/minimum inhibitory concentration ratio; AUC/MIC: area under concentration-time curve/MIC ratio.

